# ICU Delirium in Cardiac Patients

**DOI:** 10.14797/mdcvj.1246

**Published:** 2023-08-01

**Authors:** Hina Faisal, Souha Farhat, Navneet K. Grewal, Faisal N. Masud

**Affiliations:** 1 Center for Critical Care, Houston Methodist Hospital, Weill Cornell Graduate School of Medical Sciences, Houston, Texas, US; 2 Institute for Reconstructive Surgery, Houston Methodist Hospital, Weill Cornell Graduate School of Medical Sciences, Houston, Texas, US; 3 Memorial Hermann Southwest Hospital, UT Health Houston, McGovern Medical School, Houston, Texas, US

**Keywords:** ICU delirium, cardiac patients, risk factors, screening, treatment

## Abstract

Delirium is a prevalent complication in critically ill medical and surgical cardiac patients. It is associated with increased morbidity and mortality, prolonged hospitalizations, cognitive impairments, functional decline, and hospital costs. The incidence of delirium in cardiac patients varies based on the criteria used for the diagnosis, the population studied, and the type of surgery (cardiac or not cardiac). Delirium experienced when cardiac patients are in the intensive care unit (ICU) is likely preventable in most cases. While there are many protocols for recognizing and managing ICU delirium in medical and surgical cardiac patients, there is no homogeneity, nor are there established clinical guidelines.

This review provides a comprehensive overview of delirium in cardiac patients and highlights its presentation, course, risk factors, pathophysiology, and management. We define cardiac ICU patients as both medical and postoperative surgical patients with cardiac disease in the ICU. We also highlight current controversies and future considerations of innovative therapies and nonpharmacological and pharmacological management interventions. Clinicians caring for critically ill patients with cardiac disease must understand the complex syndrome of ICU delirium and recognize the impact of delirium in predicting long-term outcomes for ICU patients.

## Introduction

Delirium is a neuropsychiatric syndrome characterized by acute cognitive dysfunction and changes in attention, memory, and consciousness. According to the American Psychiatric Association’s *Diagnostic and Statistical Manual of Mental Disorders, 5th edition* (DSM-5), delirium is defined as a disturbance in attention and awareness that develops over a short period and fluctuates in severity, leading to changes in cognition, perceptual disturbances, and sleep-wake cycle disturbance.^1^

While delirium can occur in various medical conditions, including infections, drug toxicity, metabolic imbalances, and brain injury, it is prevalent in critically ill patients, particularly those in intensive care units (ICUs). Historically, the prevalence of ICU delirium is reported in 20% to 50% of patients with lower severity of illness,^2,3^ but it can be as high as 60% to 80% in mechanically ventilated patients.^4,5,6^ It is associated with increased morbidity, mortality, and long-term cognitive impairment.^4^

Despite extensive study and numerous published articles on ICU delirium, insufficient focus has been placed on the unique features of delirium affecting cardiac patients admitted to the ICU following myocardial infarction, heart failure, cardiogenic shock, and cardiac surgeries. As such, there is a paucity of data on the pharmacological therapy required for preventing and treating delirium in critically ill patients with cardiac diseases.

The high prevalence and significant impact of delirium in critically ill cardiac patients highlight the importance of prevention, early detection, and management of ICU delirium in this context. Here we provide an overview of current knowledge on the risk factors predisposing to delirium in cardiac ICU patients as well as the epidemiology, pathophysiology, clinical presentation, diagnostic tools, and appropriate disease management.

## Risk Factors for Icu Delirium in Cardiac Patients

Various risk factors can cause delirium, some similar to those predisposed to developing delirium in any other ICU setting and some specific to the cardiac ICU (CICU) setting (Table 1). The former includes nonmodifiable risk factors (such as advanced age, underlying comorbidities, namely dementia, and the degree of their severity) and modifiable (such as acute illness, medications, electrolyte disturbances, immobilization, and surgery). Advanced age is a well-established risk factor for delirium in the ICU. In fact, the aging process is associated with changes in the brain, including decreased blood flow and reduced brain volume, which can lead to cognitive impairment and, consequently, increased risk of delirium.^7^

**Table 1:** Risk factors of delirium in the cardiac intensive care unit. CICU: cardiac intensive care unit; TAVR: transcatheter aortic valve repair


**Non-modifiable**	Advanced age

	Baseline cognitive impairment

	Comorbidities

**Modifiable**	Immobilization

	Medications

	Acute illness

	Electrolyte imbalance

	Surgery

**CICU Specific**	Heart failure

	Mechanical ventilation

	Post TAVR


The likelihood of developing delirium can significantly influence the patient’s health and susceptibility. For instance, in patients with dementia and underlying comorbidities, even a mild urinary tract infection can trigger delirium.^8^ In contrast, young and healthy individuals may only experience delirium upon exposure to a series of stressors, such as general anesthesia, sleep deprivation, multiple psychoactive drugs, prolonged mechanical ventilation, and extended stay in the ICU.^9^

Risk factors that are associated explicitly with CICU patients include intraoperative and perioperative causes (medications, mechanical ventilation, transcutaneous pacing, post transcatheter aortic valve replacement, and heart failure).^10^ A 2013 study published by McPherson et al. in the *Journal of Critical Care Medicine* found that delirium is prevalent in the CICU and affects around 25% of cardiology and cardiac surgical patients. The authors found that patients who received chemical restraints via benzodiazepines or were physically immobilized with physical restraining devices were at a higher risk of developing delirium the next day.

Several other risk factors are associated with developing delirium in the CICU and cardiovascular ICU. In particular, extended periods of cardiopulmonary bypass have been linked to neurocognitive complications and delirium.^11^ The duration of bypass time is directly correlated with an increased risk of delirium, potentially due to the systemic inflammatory response, cerebral hypoperfusion, and microemboli associated with prolonged bypass.^11^ Another important risk factor is surgical complexity. Complex surgical procedures involving multiple grafts, repairs, or concomitant procedures are associated with a higher risk of delirium. The invasiveness and duration of surgery, as well as the physiological stress placed on the patient, contribute to the development of delirium.^12^

Patients who require interventions, such as placing an intra-aortic balloon pump (IABP) via the femoral artery or groin lines, may experience extended periods of limited mobility that may contribute to the development of delirium. One approach to mitigating this risk is the utilization of axillary IABPs instead of femoral ones. Axillary IABPs allow greater patient mobility and do not restrict lower limb movement.^13^ By enabling patients to mobilize earlier, this alternative approach may help reduce the risk of delirium associated with prolonged immobilization. Additionally, implementing physical therapy programs focusing on mobilizing patients with femoral catheters may be beneficial. These programs aim to encourage patients to engage in early mobilization exercises while ensuring the safety and integrity of the catheter.^12^ In coronary artery bypass graft patients, acute kidney injury has been shown to be an independent risk factor for postoperative delirium.^14^

## Pathophysiology of ICU Delirium in Cardiac Patients

Delirium is the most common complication in the general hospital setting.^15^ Despite general knowledge of the risk factors of delirium, its precise pathophysiology has yet to be fully understood. Still, it is thought to involve a complex interplay between neuroinflammation, neurotransmitter imbalances, oxidative stress, and mitochondrial dysfunction. Genetic predisposition is also a contributing factor in developing delirium in the CICU.

The inflammatory response is one of the critical factors involved in developing delirium in the CICU. Systemic inflammatory response syndrome (SIRS) is common in critically ill patients and has been associated with the development of delirium.^16^ One study examining the difference in biomarkers in patients suffering from SIRS concluded that the proinflammatory cytokine IL-8 accompanies delirium.^17^ A total of 370 serum biomarkers were identified, of which IL-6 appeared to be the most closely associated with the risk of postoperative delirium.^18^ The incidence rate of sepsis-associated delirium in the ICU is 17.7% to 31.5%,^19^ and its prognosis is similar to that of ICU delirium.^20^

Neurotransmitter imbalances are another important factor in the development of delirium. Various factors, including medications, metabolic disturbances, and inflammation, can cause these imbalances. Alterations in the levels of acetylcholine, dopamine, serotonin, and gamma-aminobutyric acid have been implicated in the pathogenesis of delirium, and drugs that affect these neurotransmitter systems have been used to treat the condition. For example, decreased acetylcholine levels have been associated with the development of delirium,^21^ while increased dopamine levels have been implicated in delirium with psychotic features.^15^ Since normal aging ultimately leads to a decrease in acetylcholine-producing cells and decreased oxidative metabolism in the brain, the low levels of acetylcholine at an advanced age puts older people at an increased risk of developing delirium.^22^

Oxidative stress is a common feature of many pathological conditions, including delirium. In the context of delirium, oxidative stress can lead to changes in neuronal function and contribute to the development of cognitive impairment. Various studies have shown that oxidative stress, whether intraoperative or postoperative, is implicated in developing cardiac diseases such as myocardial infarction, heart failure, and cardiac arrest.^23,24^

Mitochondrial dysfunction is another factor implicated in the pathogenesis of delirium. Mitochondria are the cellular organelles responsible for energy production, and dysfunction in these organelles can lead to impaired cellular function. The mitochondrial function may be impaired in critically ill patients due to decreased oxygen delivery, nutrient deprivation, or oxidative stress. It can lead to energy depletion in brain cells and disrupt normal brain function, leading to delirium.^25^

Finally, genetic factors are essential in developing delirium in the CICU. Genetic polymorphisms have been identified that are associated with an increased risk of delirium, including genes involved in inflammation, neurotransmitter function, and oxidative stress. Identifying genetic factors involved in the pathogenesis of delirium may help to identify individuals at increased risk and lead to the development of targeted therapies.^26^

## Clinical Presentation and Diagnosis of ICU Delirium in Cardiac Patients

As explained, the current criteria for diagnosis of delirium are based on the 5th edition of the DSM-5,^1^ which lists key diagnostic features including acute onset and fluctuating course of disturbance in attention, awareness, and cognition that develop over a short period.^1^ These clinical features also manifest as memory deficits, disorientation, hallucinations, fluctuating levels of alertness, and motor abnormalities.^1^ Three subtypes of ICU delirium have been described but need to be better defined—hyperactive, hypoactive, and mixed subtypes.^27^ Hypoactive delirium may be associated with the worst prognosis of these types.^28,29^

A recent study showed that hypoactive delirium accounts for 92% of delirium cases in a mixed medical and surgical cardiac ICU.^12^ Yet there are no imaging or laboratory tests to diagnose delirium; rather, it is a diagnosis of exclusion that requires careful clinical testing and observation. Although the gold standard for delirium diagnosis is evaluation by a psychiatrist using DSM-5 criteria, this is not feasible on a routine basis, making delirium very difficult to diagnose definitively in the ICU.

**Figure 1:**
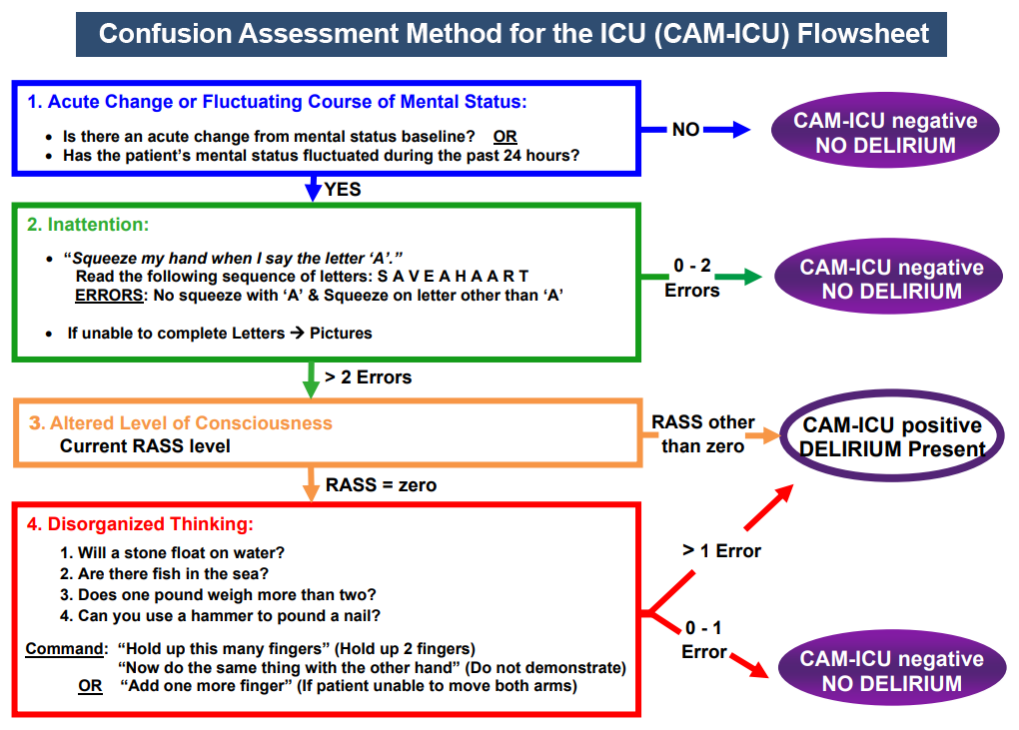
Confusion Assessment Method for the Intensive Care Unit (CAM-ICU) flowsheet. Copyright © 2002, E. Wesley Ely, MD, MPH and Vanderbilt University, all rights reserved.

Several validated screening and diagnostic tools for delirium have been developed for clinical use by various medical personnel, including the Confusion Assessment Method for the ICU (CAM-ICU),^30^ the Richmond Agitation-Sedation Scale (RAS),^31^ the Sequential Organ Failure Assessment (SOFA),^32^ and through clinical signs and symptoms of SIRS.^16^ However, ICU Pain, Agitation, and Delirium^33^ guidelines recommend two tests for the assessment of delirium in adult ICU patients: the Confusion Assessment Method for the Intensive Care Unit (CAM-ICU) (Figure 1) and the Intensive Care Delirium Screening Checklist (ICDSC).

The CAM-ICU tool assesses four features of delirium: acute onset or fluctuating course, inattention, disorganized thinking, and altered level of consciousness. The presence of acute onset or fluctuating course and inattention is necessary to diagnose delirium. The addition of sloppy thinking and an altered level of consciousness increases the diagnostic accuracy of the CAM-ICU. This tool has been shown to have high sensitivity and specificity for detecting delirium in critically ill patients, including those in the cardiac ICU.^30^

The RASS is a tool that measures the level of sedation and agitation in patients. This tool ranges from +4 (combative) to -5 (unarousable), with 0 being the target range for patients on mechanical ventilation. The RASS is useful in detecting delirium in patients in the ICU, including those in the CICU.^31^

In addition to these screening tools, clinical signs and symptoms of SIRS and SOFA scores also may be used to identify patients at risk for developing delirium. SIRS is defined as the presence of two or more of the following: fever or hypothermia, tachycardia, tachypnea, and leukocytosis or leukopenia.^34^ SOFA scores assess the degree of organ dysfunction in six systems: respiratory, coagulation, liver, cardiovascular, central nervous system, and renal. High SOFA scores have been associated with an increased risk of delirium in critically ill patients.^35^

A meta-analysis of five ICU delirium screening methods showed that the CAM-ICU and ICDSC screening tests were the most sensitive and specific tools for identifying delirium.^36^ In another systematic review and meta-analysis of nine studies evaluating CAM-ICU (including 969 patients) and four evaluating the ICDSC (including 361 patients), the pooled sensitivity of CAM-ICU was 80% (95% CI, 77-83%), with a specificity of 96% (95% CI, 95-97%) and an AUC of 0.97. The pooled sensitivity of ICDSC was 74% (95% CI, 65-82%), with a specificity of 82% (95% CI, 77-86%) and an AUC of 0.89.^37^

## Impact of ICU Delirium on Cardiac Patients

Evidence shows that delirium in critically ill cardiac patients is associated with an increased risk of cognitive decline, increased morbidity and mortality, prolonged hospital and ICU stay, and increased healthcare-associated costs. A study by Saczynski et al. found that patients who developed delirium after cardiac surgery had low mini-mental state examination scores at 1 month (24.1 vs 27.4; *P* < .001) and 1 year (25.2 vs 27.2; *P* < .001), and even at 6 months postop many had not returned to their preoperative baseline (40 vs 24%; *P* < .01).^38^ Another observational study by Pandharipande et al. reported that even at 12 months, up to one-third of critically ill patients with delirium had cognitive scores similar to those with moderate traumatic brain injury or mild Alzheimer’s disease.^39^ More importantly, the duration of delirium was noted to be an independent predictor of worse global cognition and executive function at 3 and 12 months (*P* < .05). Furthermore, beyond cognitive decline, delirium has been associated with ongoing functional decline noted at 1 month with a trend towards persistent reduction at 12 months after cardiac surgery.^40^ Quality of life also appeared worse in a prospective study with 300 cardiac surgery patients, in which delirium was associated with lower scores in seven out of eight measures of quality of life score (based on the SF-36 survey).^41^

A prospective study of 275 medical and coronary care ICUs found that delirium was noted to be associated with higher 6-month mortality (adjusted HR 3.2; 95% CI, 1.4-7.7; *P* = .008), longer hospital stay (adjusted HR 2.0; 95% CI, 1.4-3.0; *P* < .001) after adjustments for age, severity of illness, coma, and use of sedation or analgesia^6^ similar to other published data in the cardiac surgery patients.^42,43^ Moreover, studies to differentiate the attributable risk of mortality to delirium found that patients with delirium for > 2 days had a true mortality risk attributable to delirium.^44^ Furthermore, a retrospective cohort study of 6,323 patients evaluated the association between various types of delirium and 90-day mortality, and results showed that patients with mixed delirium (not hyperactive, hypoactive, or rapidly reversible) were found to have a higher 90-day mortality (1.57; 95% CI, 1.51-2.14).^45,46,47^

ICU delirium after cardiac surgery is associated with a longer duration of mechanical ventilation, higher cost, and longer hospital length of stay even when out of the ICU. One meta-analysis found that patients with delirium had a longer length of stay (standard mean difference 1.4 of 0.99 to 1.8 days; *P* < .001) compared with nondelirious patients. Additionally, the mean duration of mechanical ventilation was approximately two days longer than in patients without delirium (standard mean difference 1.8 days; *P* < .001).^48^ In a subgroup analysis within the BRAIN-ICU study, the cumulative cost secondary to higher resource utilization of ICU delirium was $17,838 (95% CI, $11,132-$23,497).^49^ Per data calculated in one study, the direct 1-year cost associated with delirium is predicted to range from $143 to $152 billion, assuming delirium occurs in 20% of the elderly patients hospitalized annually.^49,50^

## Prevention and Treatment of ICU Delirium in Cardiac Patients

Prevention and treatment of delirium involve a multidisciplinary approach, including pharmacologic and nonpharmacologic interventions such as early mobilization, environmental modifications, and delirium protocols.^33^

### Prevention

No single drug or nonpharmacologic intervention strategy has demonstrated effectiveness for delirium prevention or treatment; rather, screening, prevention, and early treatment are the mainstays of CICU delirium in cardiac patients. Several studies and clinical practice guidelines of various medical societies recommend multicomponent, nonpharmacologic strategies focused on primary prevention (ie, preventing delirium before it occurs) in patients at risk for delirium. These strategies include mobility/exercise/physical therapy, reorientation, therapeutic activities/cognitive stimulation, and sleep enhancement, with > 50% reduction in delirium across multiple studies.^51,52,53^ However, the guidelines do not provide the details on optimal implementation (eg, duration, frequency, and length of therapy of individual elements). A study by Balas et al. implemented a combined awakening and breathing coordination, delirium monitoring/management, and early exercise/mobility bundle in intubated ICU patients. This pre/post comparison reported a 50% decrease in the odds of developing delirium (OR 0.55; *P* = .03).^54^

Various studies have shown that early mobilization, exercise, and physical therapy can prevent delirium in medical and surgical ICU patients.^55,56^ In addition, early cognitive and physical therapy has been shown to reduce the incidence and duration of delirium and decrease the incidence of cognitive deficits.^57^ However, data on early cognitive exercise and interventions to prevent cognitive impairment in critically ill cardiac patients is limited. In one qualitative study, Parker et al.^58^ demonstrated many barriers to implementing early combined cognitive stimulation and goal-directed mobility performed by nurses in the medical ICU. In this study, nurses administered a workbook of evidence-based tasks to the patients at least once per shift, with tasks focused on math, alertness, problem-solving, and language. The study found low documentation (~ 30%) of the implementation of nurse-driven cognitive stimulation, which made it challenging for the study team to infer an implementation rate.

Regarding pharmacological interventions such as antipsychotics, the Best Practice Guidelines^59^ and the 2013 Society of Critical Care Medicine guidelines^33^ on pain, agitation, and delirium found insufficient evidence to recommend routine use of antipsychotics to prevent delirium; this was based on current contradictory literature and “considerable” harms of antipsychotics, in particular several cardiovascular effects, most notably QT prolongation.^60^

Sleep deprivation is another risk factor for the development of ICU delirium in patients with cardiac disease and in those who have undergone cardiac surgery. Critically ill patients have been shown to have low melatonin levels.^61^ Several trials have examined the use of melatonin and ramelteon in ICU patients with delirium, and some have shown promise. Still, these studies are limited by small sample sizes and varied methodologies, and more extensive randomized trials are needed.^55,56,62,63^

There are limited studies evaluating the role of pharmacological agents in preventing delirium in critically ill patients with cardiac disease. The DEXCOM (Dexmedetomidine Compared to Morphine) study randomized post-cardiac surgery patients to dexmedetomidine or morphine and showed that dexmedetomidine reduced the duration but not the incidence of delirium.^64^ Djaiani et al. randomized patients to dexmedetomidine versus propofol and found that dexmedetomidine reduced the incidence of delirium (17.5% vs 31.5%, OR 0.46; *P* = .028) and reduced duration (2 vs 3 days, *P* = .04).^65^ In contrast, a randomized placebo-controlled trial by Li et al. failed to demonstrate any reduction in the incidence of delirium when dexmedetomidine was administered during anesthesia and the early postoperative period (4.9% in the dexmedetomidine group vs 7.7% in the control group, *P* = .345).^66^ A recent meta-analysis that included eight randomized trials compared dexmedetomidine to propofol in post-cardiac surgery patients and demonstrated that dexmedetomidine was associated with a lower risk of delirium (RR, 0.4; 95% CI, 0.24-0.64; *P* = .0002) as well as shorter length of intubation but a higher risk of bradycardia.^67^

Optimizing mean arterial pressures based on cerebral autoregulation (estimated by transcranial Doppler) during cardiopulmonary bypass surgery has been shown to reduce the incidence of postoperative delirium.^68^ Monitoring techniques such as transcranial Doppler and cerebral oximetry with near-infrared spectroscopy (NIRS) have emerged as valuable tools for preventing and predicting postoperative cardiac delirium.^69,70,71^ Transcranial Doppler, a noninvasive method, allows for real-time assessment of cerebral blood flow velocities and provides insights into cerebral perfusion. It also helps identify cerebral hemodynamic changes that may precede the onset of delirium. By monitoring transcranial Doppler parameters such as mean flow velocity and pulsatility index, cerebral hypoperfusion or embolic events can be detected and thus facilitate prompt intervention to optimize cerebral blood flow.

Cerebral oximetry, specifically NIRS monitoring, offers another assessment dimension by measuring regional cerebral oxygen saturation (rSO_2_). This technique enables continuous noninvasive monitoring of cerebral oxygenation levels and provides valuable information about the balance between cerebral oxygen supply and demand. Decreased rSO_2_ levels may indicate compromised cerebral perfusion, potentially leading to delirium.^72^ By incorporating NIRS monitoring, clinicians can proactively detect and address cerebral oxygenation deficits, optimizing oxygen delivery to the brain and potentially reducing the risk of postoperative cardiac delirium.

### Treatment

Initial delirium treatment therapy should be focused on treating any identifiable precipitating factors, including management of hypoxia with supplemental oxygen, fluid and electrolyte imbalance corrections, pain management, maintaining perfusion by hemodynamic stability, and nutrition for hypoglycemia treatment. In a study by Fuest et al., the hemodynamic algorithm applied in high-risk non-cardiac surgery patients did not change hemodynamic interventions, improve patient hemodynamics, increase cerebral oxygenation, or change the incidence of postoperative delirium.^73^ Further management goals are aimed at reducing the severity and duration of delirium, although there is minimal data to guide the treatment of ICU delirium in cardiac patients.

Nonpharmacological strategies to reorientate the patient should include home hearing aids and glasses. There are conflicting and limited data to suggest that antipsychotics reduce the duration of delirium. In one small trial, 36 ICU patients with a diagnosis of delirium were randomized to quetiapine or placebo. The results demonstrated that patients who received quetiapine had a shorter duration of delirium (1 vs 4.5 days, *P* = .001).^74^ Another study randomized 103 patients to haloperidol, ziprasidone, or placebo and showed no difference in the duration of delirium.^75^ Based on the above evidence, the Society for Critical Care Medicine guideline on delirium provides no recommendation for using haloperidol in treating ICU delirium while stating that atypical antipsychotics may reduce the duration of delirium. If necessary, the current guideline recommends the “lowest effective dose” for the shortest duration, and only after nonpharmacological interventions have failed. It notes that these should not be used in patients at significant risk for torsades de pointes.

## Conclusion

ICU delirium in cardiac patients is a common condition that is independently associated with increased morbidity and mortality. Successful management of ICU delirium in cardiac patients requires an understanding of which patients are at the highest risk for developing delirium. A proactive approach to diagnosis and preemptive cognitive and physical stimulation can be used to prevent delirium in high-risk patients. Further studies are needed to examine the preventive strategies in critically ill cardiac patients.

## Key Points

- Delirium in cardiac patients in the intensive care unit (ICU) is a highly prevalent and preventable condition.
- ICU delirium in cardiac patients is associated with high morbidity, mortality, and cost.
- The pathophysiology of ICU delirium remains poorly understood.
- Delirium assessment and screening tools must be adopted clinically to promote widespread recognition of delirium.
- No single pharmacotherapy is available to prevent and treat ICU delirium.
- ICU clinicians should follow multicomponent, nonpharmacologic strategies focused on primary prevention.
